# Possible Role of Fish and Frogs as Paratenic Hosts of *Dracunculus medinensis*, Chad

**DOI:** 10.3201/eid2208.160043

**Published:** 2016-08

**Authors:** Mark L. Eberhard, Michael J. Yabsley, Hubert Zirimwabagabo, Henry Bishop, Christopher A. Cleveland, John C. Maerz, Robert Bringolf, Ernesto Ruiz-Tiben

**Affiliations:** Centers for Disease Control and Prevention, Atlanta, Georgia, USA (M.L. Eberhard, H. Bishop);; University of Georgia College of Veterinary Medicine, Athens, Georgia, USA (M.J. Yabsley, C.A. Cleveland);; University of Georgia Warnell School of Forestry and Natural Resources, Athens (M.J. Yabsley, C.A. Cleveland, J.C. Maerz, R. Bringolf);; Carter Center, Atlanta (H. Zirimwabagabo, E. Ruiz-Tiben)

**Keywords:** Dracunculus medinensis, tadpoles, paratenic hosts, Guinea worm disease, dracunculiasis, copepods, Chad, parasites, nematodes, roundworms

## Abstract

Copepods infected with *Dracunculus medinensis* larvae collected from infected dogs in Chad were fed to 2 species of fish and tadpoles. Although they readily ingested copepods, neither species of fish, Nile tilapia (*Oreochromis niloticus*) nor fathead minnow (*Pimephalis promelas*), were found to harbor *Dracunculus* larvae when examined 2–3 weeks later. Tadpoles ingested copepods much more slowly; however, upon examination at the same time interval, tadpoles of green frogs (*Lithobates* [*Rana*] *clamitans*) were found to harbor small numbers of *Dracunculus* larvae. Two ferrets (*Mustela putorius furo*) were fed fish or tadpoles that had been exposed to infected copepods. Only the ferret fed tadpoles harbored developing *Dracunculus* larvae at necropsy 70–80 days postexposure. These observations confirm that *D. medinensis*, like other species in the genus *Dracunculus*, can readily survive and remain infective in potential paratenic hosts, especially tadpoles.

## Abstract 2

Tadpoles fed infected copepods can harbor infective *D. medinensis* larvae and thus serve as potential paratenic hosts.

The global campaign to eradicate dracunculiasis (or Guinea worm disease [GWD]), which began at the Centers for Disease Control and Prevention (CDC) in 1980 and has been led by the Carter Center since 1986, has assisted 17 of 21 affected countries to interrupt transmission. In 1986, an estimated 3.5 million cases occurred annually in 20 countries (the separation of Sudan and South Sudan created a 21st country in 2011). Today, only 4 countries (Chad, Ethiopia, Mali, and South Sudan) still have endemic transmission of GWD. In 2014, only 126 cases were reported from these 4 countries, and only 22 cases were reported during January–November 2015 (*1*). Since the beginning of the campaign, all interventions against GWD have focused on preventing contamination of stagnant sources of drinking water by patients with patent GWD and preventing ingestion of infected copepods. However, in 2013, several unusual epidemiologic findings were noted in Chad, including absence of disease outbreaks associated with sources of drinking water common to many residents year-to-year in affected villages and a relatively common infection of domestic dogs with Guinea worms genetically indistinguishable from those from human cases. This finding led to the hypothesis that an aquatic paratenic host (an intermediate host that serves as transport host for parasite larvae) was involved in the transmission of *Dracunculus medinensis* in Chad (*2*). Since 2013, the sporadic pattern of human cases in Chad has continued, the number of infections in dogs has continued to increase, and the presence of a paratenic host in the transmission cycle seems more likely.

The purpose of our study was to expose, under laboratory conditions, 2 species of fish and 2 species of tadpoles to *D. medinensis*–infected copepods, in an effort to determine whether these species would support viable larvae (and thus serve as a paratenic host). These potential paratenic hosts were then fed to an experimental definitive host (a domestic ferret) to determine if any third-stage larvae (L3) present would undergo further development in ferrets.

## Materials and Methods

During July 20–22, 2015, batches of first-stage larvae (L1) were recovered from 5 Guinea worms removed from infected dogs resident in villages within the dracunculiasis-endemic zone along the Chari River between the cities of Guelendeng and Bousso in the Mayo-Kebbi Est region of Chad. Copepods were collected locally in the Guelendeng area (dracunculiasis-endemic zone) and exposed to the L1 per standard methods (*3*). L1 were mixed with copepods in a ratio of ≈3–5:1 to ensure a high rate of infection but not high enough to cause copepod mortality. Infected copepods were maintained for 5–7 days in 1.5-L water bottles and then transported to laboratories at CDC (Atlanta, GA, USA), where they were maintained in culture per standard practices (*3*). Beginning at day 12 after exposure, copepods were dissected to determine level of infection and stage of development of larvae. Many larvae observed at day 12 were early (and infective) L3. To allow full maturation of the infective larvae, no copepods were used in experimental studies before day 14.

For experimental trials, 2 species of fish (29 Nile tilapia [*Oreochromis niloticus*] and 36 fathead minnows [*Pimephalis promelas*]) and 2 species of tadpoles (20 Fowler’s toads [*Bufo fowleri*] and 10 green frogs [*Lithobates* (*Rana*) *clamitans*]) were used. Both fish species were captive-raised and the tadpoles were wild-caught (green frogs raised from egg masses in captivity) in Georgia. Because the toads and some of the ranid tadpoles were very young at the time of exposure, species identity was confirmed by amplification and sequencing of a ≈500-bp region of the 16S rRNA gene with primers Amph-sp-F (5′-CTGTTTACCAAAAACATCG-3′) and Ecto-univ-R (5′-ATCCAACATCGAGGTCGT-3′). Sequences were a 100% match to GenBank accession numbers AY680224 (toads) and DQ283185 (ranids) confirming their identity. Finally, 2 age classes of green frog tadpoles were used. 

Beginning at day 14 after exposure to *D. medinensis* L1, batches of infected copepods were fed to groups of fish or tadpoles. Fish and tadpoles, separated by species, were exposed to copepods in shallow water in a 500-mL beaker with a bubbling air source. Both fish species immediately began to ingest copepods, all of which had been ingested by 24 hours. In contrast, tadpoles ingested copepods more slowly, and some copepods remained uneaten after 3 days of exposure.

Beginning 1 week after exposure, groups of 4–5 fish and tadpoles were examined for *Dracunculus* larvae. Fish and tadpoles were euthanized by overdose of pH-neutral buffered tricaine methane sulfonate (MS-222), followed by decapitation, and then examined grossly under a dissecting scope to see if any larvae could be visualized in fin or tail fin or under skin. Then, the entire body, including head and viscera, was dissected to fully separate all tissues, and macerated tissues were examined under a dissecting microscope. If no larvae were observed, the tissues were allowed to sit in phosphate-buffered saline for 1–2 hours and reexamined under a dissecting or compound microscope. A total of 19 tilapia, 19 fathead minnows, 11 *Bufo* tadpoles, and 9 *Lithobates* tadpoles were examined by dissection. For a subset of 6 fish (3 of each type), tissues were grossly macerated, examined microscopically, and then digested in a 0.5% pepsin solution for 1 hour before examination for larvae.

Two colony-reared ferrets (*Mustela putorius furo*) were fed either tadpoles or fish mixed in canned cat food to determine infectivity of L3 from aquatic hosts. All dissected fish and the additional 5 tilapia and 11 fathead minnows that were not dissected were fed to 1 ferret. The other ferret was fed all dissected tadpoles and an additional 9 *Bufo* and 3 *Lithobates* tadpoles that had not been dissected. Ferrets were euthanized and examined at 70–83 days after exposure to fish or tadpoles. Animals were examined per previously proven methods for detecting developing *Dracunculus* (*3*). All animal procedures were reviewed and approved by the University of Georgia Institutional Animal Care and Use Committee (no. A2014 11–010).

## Results

No *Dracunculus* larvae were detected in dissected fish or *Bufo* tadpoles, but 4 of 7 *Lithobates* tadpoles were infected with 1–3 *Dracunculus* L3. Infections were noted in both age classes. The exact locations of the larvae were not determined, but they were recovered from the body musculature or head. No larvae were observed in the body cavity or viscera. The larvae recovered from tadpoles were slightly larger and more active than L3 recovered from copepods.

No worms were detected in the ferret that had been fed fish, but in the ferret that had ingested tadpoles, 3 young developing worms were recovered. All 3 worms were females and measured 1.4, 2.0, and 2.7 cm in length by 295–350 μm in maximum diameter. Two of the worms were recovered from the right hind leg, and the third worm was recovered from the lower left abdominal wall. The largest worm was coiled under the muscle fascia, whereas the other 2 worms were present in adipose tissue.

## Discussion

These results, although limited in scope, clearly confirm that *D. medinensis*, like *D. insignis*, can use an aquatic paratenic host; specifically, at least 2 species of amphibians (*4*–*6*). Although tadpoles consumed far fewer copepods, most tadpoles exposed to infected copepods subsequently had infections, which is consistent with previous data that showed a high percentage of adult frogs (*L. pipiens* and *L. clamitans*) acquired infections when a very high dose of infected copepods (n = 200–500) were given by mouth (*4*).

*D. insignis* larvae recovered from tadpoles previously were stated to be slightly larger and more active than infective larvae recovered from copepods, which is what we noted in our study with *D. medinensis* (*4*,*6*), in which the larvae grew ≈20% after 15–18 days. Previously these *D. insignis* larvae recovered from tadpoles or frogs were infective to a single raccoon or ferrets, respectively, proving that these larger larvae are infectious (*4*,*5*). Similarly, the larger *D. medinensis* larvae we recovered from tadpoles were infectious for a domestic ferret.

The absence of larvae in the 2 species of fish we included in this study does not rule out a role of fish as paratenic hosts. Previously, a low percentage of fish exposed to L3 recovered from copepods subsequently had infections (*4*). Although sample sizes were low, 2 of 4 white suckers (*Catostomus commersonii*) and 1 of 2 rainbow trout (*Oncorhynchus mykiss*) had 1–2 larvae recovered after exposure to 100–180 L3. Thus, we may not have exposed our fish to enough larvae to become infected (*4*). However, fish species variability in susceptibility is probable, given that common shiners (*Luxilus cornutus*) failed to become infected even though 3 were exposed to >200 larvae (*4*). Future studies should investigate the fate of *Dracunculus*-infected copepods after ingestion by fish hosts, dose required to establish infections in fish, and additional trials with other species of fish (e.g., *Gambusia* [mosquito fish]) that are known to predate copepods. Tilapia, 1 type of fish used in this study, is common in Chad and widely used as a food source. However, many other types of fish are present in Chad, and continued study of tilapia and other native fish should also be undertaken before ruling out fish as potential paratenic hosts.

Collectively, our data and the findings of previous reports indicate that *Dracunculus* larvae in general, and *D. medinensis* larvae specifically, are well-adapted to using a paratenic host and that tadpoles of *Lithobates* (*Rana*) and *Xenopus* species are appropriate hosts (*4*,*5*). *Lithobates* spp. are members of the family Ranidae, which has a near global distribution that includes more than 180 known species in sub-Saharan Africa (*7*). This study also confirms that domestic ferrets, like domestic cats, domestic dogs, and monkeys, can serve as experimental definitive hosts for *D. medinensis*. The recovered worms were of consistent size and development as previously reported for worms of similar age recovered from dogs or monkeys (*8*,*9*). Finally, these results also suggest that a more extensive examination of tadpoles and frogs in Chad is warranted. Although a small number (n = 28) of ranid frogs from Chad were previously examined for *Dracunculus* larvae and all were negative (*2*), sample sizes were low. Because natural infections of tadpoles or frogs have not been documented for either *D. medinensis* or *D. insignis,* the prevalence of natural infections is unknown; therefore, larger numbers of wild-caught frogs should be examined in future efforts. In addition to identifying which aquatic animals are acting as paratenic hosts for *D*. *medinensis* in Chad, it would be important to also identify which wild animals are predators of these transport hosts and whether those predators develop patent infections, thus helping maintain transmission of the parasite.
